# Structural Analysis of the Large Stokes Shift Red Fluorescent Protein tKeima

**DOI:** 10.3390/molecules29112579

**Published:** 2024-05-30

**Authors:** Ki Hyun Nam, Yongbin Xu

**Affiliations:** 1 College of General Education, Kookmin University, Seoul 02707, Republic of Korea; 2 Department of Bioengineering, College of Life Science, Dalian Minzu University, Dalian 116600, China; 3 Key Laboratory of Biotechnology and Bioresources Utilization of Ministry of Education, College of Life Science, Dalian Minzu University, Dalian 116600, China

**Keywords:** tKeima, large Stoke shift, Keima, fluorescent protein, structure

## Abstract

The Keima family comprises large Stokes shift fluorescent proteins that are useful for dual-color fluorescence cross-correlation spectroscopy and multicolor imaging. The tKeima is a tetrameric large Stokes shift fluorescent protein and serves as the ancestor fluorescent protein for both dKeima and mKeima. The spectroscopic properties of tKeima have been previously reported; however, its structural basis and molecular properties have not yet been elucidated. In this study, we present the crystallographic results of the large Stokes shift fluorescent protein tKeima. The purified tKeima protein spontaneously crystallized after purification without further crystallization. The crystal structure of tKeima was determined at 3.0 Å resolution, revealing a β-barrel fold containing the Gln-Tyr-Gly chromophores mainly with cis-conformation. The tetrameric interfaces of tKeima were stabilized by numerous hydrogen bonds and salt–bridge interactions. These key residues distinguish the substituted residues in dKeima and mKeima. The key structure-based residues involved in the tetramer formation of tKeima provide insights into the generation of a new type of monomeric mKeima. This structural analysis expands our knowledge of the Keima family and provides insights into its protein engineering.

## 1. Introduction

Fluorescent proteins (FPs) are widely used as optical probes in molecular and cell biology studies such as Förster or fluorescence resonance energy transfer (FRET) [1], optogenetics [2,3], chemogenetics [4,5], subcellular localization [6,7], in vivo imaging [8,9], and genome editing [10,11,12]. Additionally, it has been proposed as a material for developing metal biosensors and pH indicators, capitalizing on the characteristic fluorescence emission intensity changes in FPs triggered by external stimuli, such as pH or metal ions [13,14,15,16,17]. Although various fluorescent proteins have been developed, new fluorescent proteins are continuously being developed, or existing FPs are being engineered because of the need for more sophisticated FPs. Furthermore, although the optical properties of FP vary depending on the purpose of the research, photostability, red-shifted emission, high fluorescence quantum yield, a high extinction coefficient (ε), and a large Stokes shift (≥100 nm) are generally considered important factors [18,19,20,21].

Among the properties of FP, its large Stokes shift (LSS) is advantageous for fluorescence imaging applications as self-absorption is diminished owing to the wide gap between the excitation and emission maxima [22,23]. Their optical properties minimize the inner filter effect and reduce potential crosstalk between multiple emitters [22,23]. Accordingly, LSS FPs are useful in multicolor imaging experiments, two-photon laser scanning microscopy (TPLSM), dual-color fluorescence cross-correlation spectroscopy (FCSS) for quantifying protein–protein interactions, fluorescence resonance energy transfer (FRET), and multicolor imaging [23,24,25,26].

Various LLS FPs, such as LSSmKate [27], LSSm-Orange [28], mBeRFP [29], and the Keima family [23], have been reported. Keima was developed using a nonfluorescent protein from the stony coral *Montipora* sp. through semi-random mutagenesis [23]. The maximum absorbance and far-red fluorescence emission of Keima were observed at 440 nm and 616 nm, respectively. The name “Keima” is derived from a shogi (Japanese chess) piece that jumps around like a chess knight due to its large Stokes shift characteristics [23]. Analytical equilibrium ultracentrifugation analysis showed that the absolute molecular mass of Keima was approximately 106 kDa, which is four times larger than the primary structure of the protein, indicating that Keima forms a homotetrameric complex, “tKeima” [23], and tKeima has a chromophore consisting of a Gln-Tyr-Gly tripeptide. The molar extinction coefficient (ε) and fluorescence quantum yield (Φ) of tKeima at pH 7.4 were found to be 14,500 M^−1^ cm^−1^ (at 440 nm) and 0.22, respectively [23]. Tetrameric FP does not limit its use as an optical marker of gene expression [30]. However, tetrameric FP interferes with maturation and FRET between the subunits [31]. Furthermore, the aggregation of FP may hinder all possible applications because of their considerable cellular toxicity [30]. The tKeima was also engineered into dimeric (dKeima) or monomeric Keima (mKeima). The λ_ex_/λ_em_, ε, and Φ of dKeima were 440/616 nm, 24,600 M^−1^ cm⁻^1^, and 0.31, respectively [23]. The λ_ex_/λ_em_, ε, and Φ of mKeima were 440/620 nm, 14,400 M⁻^1^ cm⁻^1^, and 0.24, respectively [23]. Although the spectroscopic characteristics and applications of the Keima family have been studied, the structural characteristics of tKeima, which are the origins of mKeima and dKeima, have not yet been reported.

To better understand the molecular properties of the Keima family, here a crystallographic study of the large Stokes shift FP tKeima was reported. Purified tKeima spontaneously crystallized, and its crystal structure was determined at 3.0 Å resolution. The structural properties of the tetrameric formation and the chromophore environment of tKeima were analyzed. The structural properties of tKeima were compared with those of mKeima and dKeima. These results will help the molecular functions of tKeima and provide insights into the engineering of FPs.

## 2. Results

### 2.1. Spontaneously Grown tKeima Crystal

**Figure 1:**
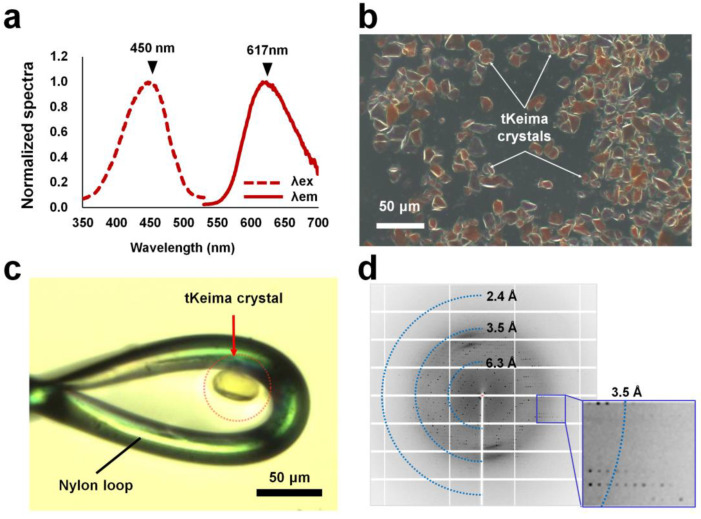
Spectroscopic and crystallographic analysis of tKeima. (**a**) Spectroscopic analysis of purified tKeima. The maximum excitation and emission peaks of tKeima were at 450 and 617 nm, respectively. (**b**) Microscopic view of spontaneously grown tKeima crystals. (**c**) The tKeima crystal was used for diffraction data collection. (**d**) Diffraction pattern of the spontaneously grown tKeima crystal.

Condon-optimized tKeima was overexpressed in *E. coli* and showed a tetrameric state through gel filtration chromatography. Spectroscopic analysis showed that purified tKeima exhibited maximum excitation and emission peaks at 450 and 617 nm, respectively (Figure 1a). The fluorescence emission peak of tKeima in this experiment was identical to that reported previously; however, the excitation peak of tKeima in this experiment was blue-shifted by 10 nm [23]. Purified tKeima was concentrated for crystallization and was stored at 4 °C before crystallization. Interestingly, the concentrated tKeima proteins spontaneously crystallized in microtubes the following day (Figure 1b). Most of the tKeima crystals were irregularly shaped with sizes between approximately 10 and 50 μm (Figure 1b). For diffraction data collection, tKeima crystals were immersed in a cryoprotectant solution containing 10 mM Tris–HCl (pH 8.0), 200 mM NaCl, and 20% (*v*/*v*) glycerol. The diffraction intensities and limits varied depending on the crystal size and shape. Among the differently shaped tKeima crystals, rhombic-shaped tKeima crystals with dimensions of approximately 20 × 20 × 40 μm^3^ were found and used for data collection (Figure 1c). This tKeima crystal diffracted up to 3.0 Å (Figure 1d).

### 2.2. Overall Structure

The tKeima crystal belongs to the orthorhombic space group P22_1_2_1_; the cell dimensions were a = 69.88 Å, b = 83.50 Å, and c = 109.78 Å, occupying two tKeima molecules in an asymmetric unit (Table 1). The final model structure was refined at 3.0 Å, with R_work_ and R_free_ values of 19.6 and 24.7%, respectively. The electron density maps of tKeima were well defined for the Met1-Gly222 residues, including the three additional N-terminals (Ser–His–Met).

**Figure 2:**
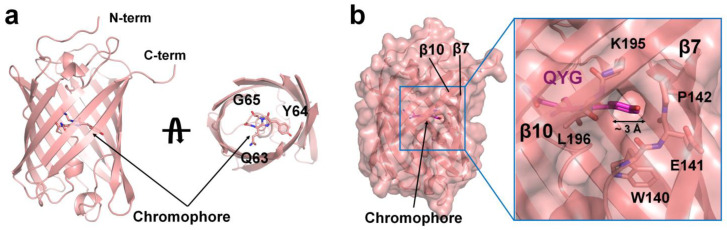
Crystal structure of tKeima. (**a**) Cartoon representation of the tKeima monomer. The chromophore consisting of the Gln-Tyr-Gly tripeptide located at the center of the β-barrel. (**b**) Surface structure of tKeima. A solvent-accessible hole was observed between β7 and β10 strands of the β-barrel.

The tKeima monomer had a β-barrel fold with 11 β-strands (Figure 2a). The chromophore of tKeima was located almost at the center of the β-barrel and contained a tripeptide Gln63-Tyr64-Gly65 that showed p-hydroxybenzylidineimidazolinone through posttranslational modification (Figure 2a). The superposition of two tKeima molecules in an asymmetrical unit showed high similarity with an r.m.s. deviation of 0.213 Å. The surface structure of tKeima showed a hole with a diameter of approximately 3 Å between the β7 and β10 strands of the two tKeima molecules within the asymmetric unit (Figure 2b). The depth from the hole entrance to the tyrosine hydroxyl group of the chromophore was approximately 6.5 Å. This hole allows for the direct observation of the hydroxyl group of the tyrosine ring of the chromophore. The hydroxyl group of the chromophore was involved in protonation/deprotonation [32,33]. Therefore, this hole, which is solvent-accessible, can directly affect the fluorescence properties of the chromophore, depending on the environmental solution. No other solvent-accessible holes were observed in the surface structure of tKeima. The water molecule in the vicinity of the chromophore was important for stabilizing the chromophore and its fluorescence properties [34]. However, the water molecule was not defined in the current model structure of tKeima. To better understand the water-mediated molecular function of tKeima, the determination of its high-resolution structure is necessary.

### 2.3. The tKeima Chromophore

**Figure 3:**
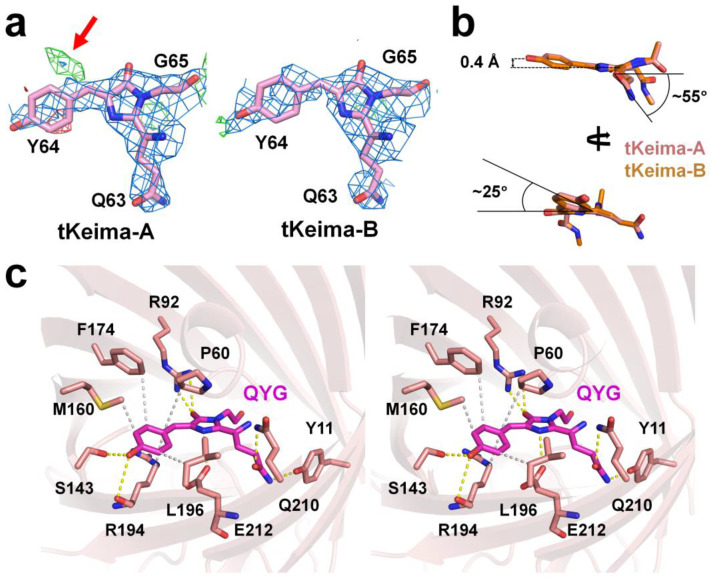
The tKeima chromophore and its environment. (**a**) Electron density maps of 2mFo-DFc (blue, 1.0 σ) and mFo-DFc (green, 3.0 σ and red, −3.0 σ) of the tKeima chromophore. The trans-conformation of the chromophore is indicated by the red arrow. (**b**) Analysis of the tKeima chromophore. (**c**) Stereo view of the tKeima chromophore and its interacting residues.

The electron density maps of the imidazoline ring of the tKeima chromophore and its neighboring residues in the vicinity of the chromophore were clear, whereas the electron density map of the tyrosine ring group region of the tKeima chromophore was relatively poor (Figure 3a). In tKeima-A, the chromophore exhibited a cis-conformation of the electron density of the tyrosine ring group of the chromophore. After refinement, a partially positive mFo-DFc electron density map was observed at a position corresponding to the trans-configuration (Figure 3a). This tentatively indicates that there may be some trans-conformations in the chromophore of tKeima (see Section 3). In tKeima-B, the electron density map of the chromophore showed a cis-conformation, whereas no electron density map was observed at the position corresponding to the trans-conformation. These results indicate that the tKeima chromophore mainly shows the cis-conformation between the tyrosine and imidazoline rings; however, the tyrosine ring group of the chromophore has a relatively flexible conformation compared to the surrounding amino acids rather than a rigid conformation inside the β-barrel.

The model building results showed that the two tKeima chromophores in the asymmetric unit showed a cis-configuration, and the superimposition of these two chromophores showed an almost identical configuration (Figure 3b). The orthorhombic view showed that the tKeima chromophore was nonplanar (Figure 3b). Based on the imidazoline ring of the chromophore, the tyrosine ring group was shifted by approximately 0.4 Å in the opposite direction of the Ser143 residue, and the tyrosine ring was rotated in the clockwise direction by approximately 25° (Figure 3b). The side chain of Gln in the tKeima chromophore rotated clockwise by approximately 55°, which was observed from the side view of the imidazoline ring (Figure 3b).

A chromophore exhibits unique fluorescence characteristics not only by sequence but also by interactions with the surrounding amino acids [35,36]. The tKeima chromophore maintained various interactions with the surrounding amino acids (Figure 3c and Table 2). The hydroxyl group of the tyrosine ring of the tKeima chromophore interacts with the OG atom of Ser143 at a distance of 2.98 Å (average distance from two tKeima molecules of an asymmetric unit) (Figure 3c). Furthermore, the tyrosine ring was surrounded by the residues of the CB atom of Pro60, the CD atom of Met94, the CE atom of Met160, the CE2 atom of Phe174, and the CB atom of Leu196 residues at distances of 4.33, 3.46, 3.24, 4.55, and 3.42 Å, respectively (Figure 3c). The O2 atom of the imidazoline ring of the QYG chromophore interacts with the NH1 and NH2 atoms of Arg92 at distances of 3.45 and 2.74 Å, respectively (Figure 3c). The NE1 atom of the Gln side chain of QYG interacted with the hydroxyl group of Tyr11 at a distance of 3.26 Å. The OE1 atom of the Gln side chain of QYG interacted with the NE2 atom of Gln210 at a distance of 3.18 Å (Figure 3c). Overall, the position of the imidazoline ring of the tKeima chromophore was stabilized by hydrogen bonds. Moreover, the position of the tyrosine ring group of tKeima was stabilized by hydrogen bonds and hydrophobic interactions.

### 2.4. Tetrameric Structure of tKeima

**Figure 4:**
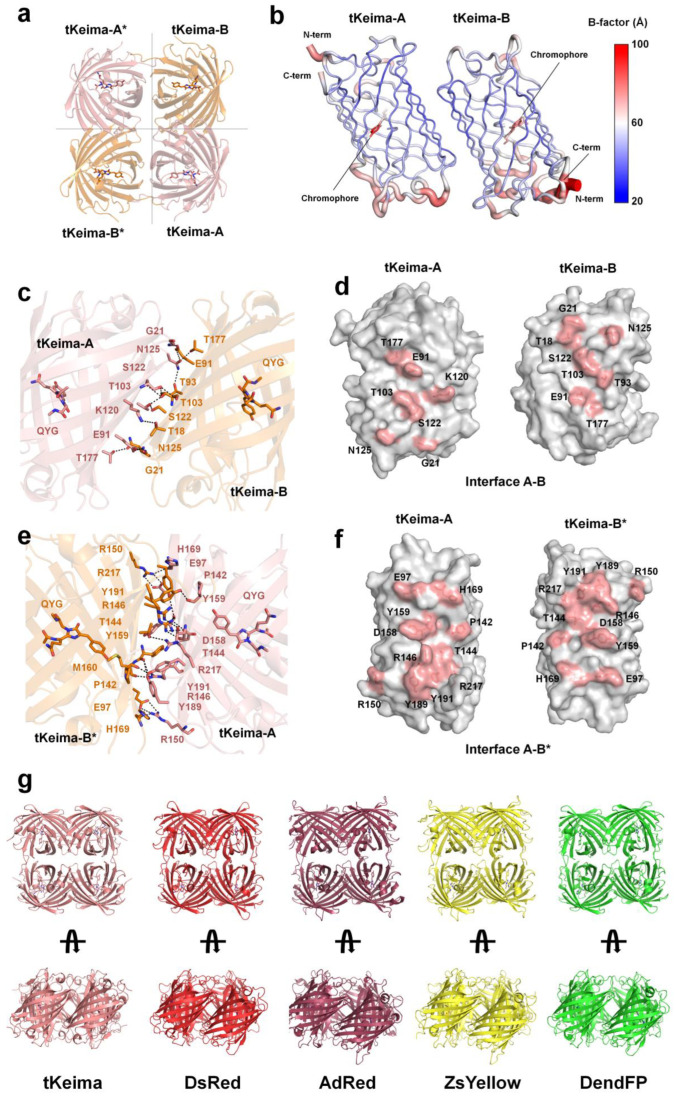
Structural analysis of tetrameric tKeima. (**a**) Tetrameric arrangement of tKeima with 222 symmetry. (**b**) B-factor putty representation of tKeima. (**c**) Close-up and (**d**) open-book view of the interaction residues of interface A–B of tKeima. Hydrogen bonds and salt bridges are indicated by the dotted line. (**e**) Close-up and (**f**) open-book view of the interaction residues of interfaces A–B* of tKeima. (**g**) Structural comparison of the tetrameric assembly of tKeima, DsRed (PDB code: 1GGX), AdRed (6AA7), ZsYellow (6LOF), and DendFP (7DIG).

Two tKeima molecules were occupied in the asymmetric unit, and the 222 symmetry of the crystal packing represented tetramer formation (Figure 4a). This crystallographic result is consistent with the formation observed in solution through gel filtration chromatography, as well as previous reports of tetrameric tKeima confirmed by analytical equilibrium ultracentrifugation analysis [23]. In the tKeima structure, the interfaces of molecules A–B and A*–B* were equivalent, and those of A–B* and A*–B were equivalent (Figure 4a). The B-factor analysis showed that the tetramer interface area of tKeima was rigid, whereas the top and bottom of the β-barrel was relatively flexible (Figure 4b).

The surface areas of molecules A and B were approximately 10,390 Å^2^ and 10,166 Å^2^, respectively. At the A–B interface of tKeima, the buried surface area was ~717 Å^2^, accounting for 7.0% of the total surface area (Figure 4c,d). The A–B interface is mainly stabilized by nine hydrogen bonds (Glu91-Asn125, Thr103-Thr103, Thr103-Ser122, Ser122-Thr103, Asn125-Glu91, Asn125-Thr93, Asn125-Thr177, and Thr177-Asn125) on the surface of the tKeima molecules (Figure 4b and Table 3). The theoretically calculated solvation energy gain at the complexation of interface A–B was −5.38 kJ/mol. At the A–B* interface of tKeima, the buried surface area was approximately 1260 Å^2^, accounting for approximately 12% of the total monomer surface area (Figure 4e,f). The A–B * interface is stabilized by 14 hydrogen bond (molecules A–B*: Glu97-Arg150, Pro142-Tyr191, Thr144-Arg146, Arg146-Met160, Arg150-Glu97, Asp158-Arg146, Tyr159-Arg146, His169-Arg150, Tyr189-Tyr159, and Tyr191-Pro142) and seven salt bridge (Glu97-Arg150, Arg150-Glu97, and Asp158-Arg146) interactions (Figure 4e and Table 3). The theoretically calculated solvation energy gain at the complexation of interface A–B* was −22.50 kJ/mol. Thus, the tetrameric interfaces of tKeima were stabilized by hydrogen bonds and salt–bridge interactions. The A–B* interface involves a greater number of amino acids distributed over a wider area than the A–B interface, resulting in more extensive interactions with low solvation energy.

The tetrameric formation of tKeima was compared with previously reported tetrameric FPs such as DsRed (PDB code: 1GGX) [37], AdRed (6AA7) [38], ZsYellow (6LOF) [13], and DendFP (7DIG) [39]. The sequence identities of tKeima with DsRed, AdRed, ZsYellow, and DendFP were 66.7%, 42.12%, 42.82%, and 60.9%, respectively. The overall assembly of the FP tetrameric formation was similar (Figure 4g). The superimposition of tKeima with tetrameric DsRed, AdRed, ZsYellow, and DendFP showed r.m.s. deviations of 1.76, 2.11, 2.31, and 1.22 Å, respectively. These results indicate that although the sequence and spectroscopic properties of tKeima differ from those of DsRed, AdRed, ZsYellow, and DendFP, the properties of tetrameric formation are evolutionarily conserved.

### 2.5. Structural Comparison of the Keima Family

**Figure 5:**
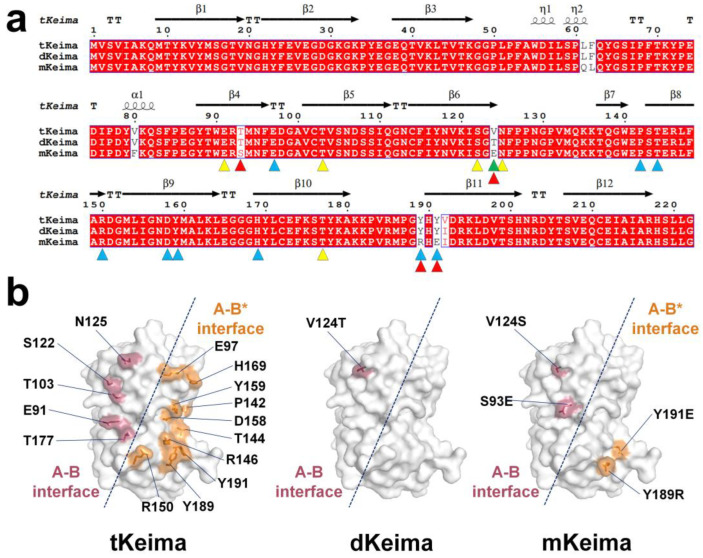
A comparison of the amino acids and structures of the Keima family. (**a**) Structure-based sequence alignment of tKeima (UniProt: Q1JV72), dKeima (Q1JV71), and mKeima (Q1JV70). The residues involved in the oligomer states of tKeima are indicated using yellow (A–B interface) and blue (A–B* interface) triangles. The mutation sites located on the surface of dKeima and mKeima are indicated by green and red triangles, respectively. (**b**) Surface structure of key residue involved in the tetrameric interface of tKeima and mutation sites located on the surface of dKeima and mKeima.

To better understand the structural properties of the Keima family, the amino acid sequence and structure of tKeima were compared to those of dKeima and mKeima. A dimeric form of dKeima was created by substituting two amino acids (V124T and V192I) from the tetrameric tKeima [23]. Seven amino acids (L61Q, F62L, V80F, T93S, T124E, Y189R, and Y191E) in dimeric dKeima were substituted to generate monomeric mKeima [23]. Furthermore, tKeima showed sequence identities of 99.1% and 96.4% with dKeima and mKeima, respectively (Figure 5a).

The tetrameric interface of tKeima is stabilized by 17 residues. In tKeima molecule A, 5 amino acids (Glu91, Thr103, Ser122, Asn125, and Thr177) contribute to stabilizing the A–B interface, whereas 10 amino acids (Glu97, Pro142, Thr144, Arg146, Arg150, Asp158, Tyr159, His169, Tyr189, and Tyr191) are involved in stabilizing the A–B* interface during the tetrameric formation of tKeima (Figure 5b).

At two mutation sites (V124T and V192I) in dKeima, the position Val124 is located between the A–B interface of tKeima (Figure 5b), while the Val192 side chain is located inside the β-barrel. Consequently, the position and molecular properties of Val124 are crucial for disrupting tKeima into dKeima, whereas Val192 may not affect the disruption of tKeima into dKeima. In the seven mutation sites (L61Q, F62L, V80F, T93S, T124E, Y189R, and Y191E) in mKeima, Thr93, Thr124, Tyr189, and Tyr191 are found in the tetrameric interface of tKeima (Figure 5b), while the Leu61, Phe62, and Val80 side chain residues are located inside the β-barrel. Consequently, Thr93, Thr124, Tyr189, and Tyr191 correspond to the key residues that disrupt the interface of tKeima to generate mKeima. Leu61, Phe62, and Val80 did not affect mKeima generation. Upon analysis of the tetrameric interface of tKeima, it was determined that Thr93 and Val124 could interfere with the interaction of the A–B interface, whereas Tyr189 and Tyr191 could interfere with the interaction of the A–B* interface. The positions of the Val124 or Ser93 amino acids that were mutated in dKeima and mKeima to disrupt the A–B interface in tKiema were not identical to the A–B interface interaction residues in tKeima. The positions of Tyr189 and Tyr191, which were mutated to disrupt the A–B* interface in mKeima, were consistent with those of the A–B* interface in tKeima.

## 3. Discussion

FPs with a large Stokes shift are widely used in multicolor imaging experiments. Among these, the Keima family exhibits a wide gap between the excitation and emission peaks, minimizing the inner-filter effect and reducing the potential crosstalk between multiple emitters. Biologically, the original Keima has a tetrameric formation; however, experimentally, a monomeric mKeima is preferred. Although mKeima is preferred for applications, it is essential to understand the overall characteristics of tKeima to gain insight into the development of improved large Stoke shift FPs in the future. In this study, the crystal structure of tKeima is reported for the first time, and its detailed structural characteristics are described, which can be used to further study the Keima family.

The purified tKeima protein underwent spontaneous crystallization, and the crystals exhibited variations in shape and size with corresponding differences in X-ray diffraction intensities. The crystal structure was determined using one of the crystals at a resolution of 3.0 Å. To obtain a higher-resolution crystal structure, protein crystallization should be attempted before spontaneous crystal growth to potentially provide more accurate insights into the flexibility or conformation of the chromophore.

The tKeima exhibits a typical β-barrel fold, and the electron density map of the imidazoline ring of the chromophore was found to be clear; however, the tyrosine ring region was relatively disordered. Furthermore, molecule A of tKeima displays a partial positive electron density in the trans-conformation, tentatively implying flexibility or potential for both the cis- and trans-conformations in the tyrosine portion of the chromophore of tKeima. Two independent research groups deposited four crystal structures of mKeima (PDB codes 2WHS, 2WHT, 2WHU, and 3IR8) in the Protein Data Bank. Violot et al. reported the crystal structure of mKeima at pH 3.8 (PDB code: 2WHS), pH 5.6 (2WHT), and pH 8.0 (2WHU), showing the cis, cis–trans mixing, and trans-configurations of the chromophore, respectively [40]. This suggests that the configuration of the chromophore may vary with pH. Specifically, the observation of both cis- and trans-conformations of the chromophore at pH 5.6 tentatively indicates that the chromophore may exhibit two conformations at a single pH position. Meanwhile, Henderson et al. reported the crystal structure of mKeima at pH 7.0 (PDB code: 3IR8), revealing the cis-configuration of the mKeima chromophore [41]. This finding is inconsistent considering the tendency of the chromophore configuration to change with pH, as observed in a previous study. This suggests that factors other than pH may influence chromophore configuration, although these factors have not yet been reported. In this study, tKeima was observed to crystallize at pH 8, and the electron density indicated a preference for the cis-configuration in the chromophore. However, as mentioned earlier, an additional mFo-DFc electron density map was observed in the upper region of the tyrosine ring in the chromophore of tKeima, suggesting the tentative possibility that some tKeima chromophores may adopt a trans-conformation. When the cis-conformation of the tKeima chromophore changes to a trans-conformation, the bond between the tyrosine and imidazole rings of the chromophore rotates by 180°, and the tyrosine ring of the chromophore in the trans-conformation may affect residues in the vicinity of the chromophore. To address this, high-resolution crystal structures at various pH levels should be determined in future studies.

Based on tKeima structure analysis, we identified the key residues that stabilize the tetrameric interface. Based on these findings, it was possible to distinguish between interface-disrupted amino acids and amino acids unrelated to the interface in the mutated residues of dKeima and mKeima. Although the mKeima mutation site does not match with the A–B interface of tKeima, it matches with the A–B* interface of tKeima. Additionally, along with the amino acid positions that were mutated in mKeima, we found that various amino acids stabilized the tetrameric interface in tKeima. This suggests that the results of the tetrameric interface analysis of tKeima can provide information for the generation of a new type of monomeric Keima, distinct from the previously engineered dKeima and mKeima. As this study provides structural information on the tetrameric interface of tKeima, the generation of monomeric Keima by amino acid substitution is feasible based on this structural information. However, it is crucial to note that substituting amino acids into the FP can alter its fluorescence characteristics, potentially improving or deteriorating the spectroscopic properties of tKeima. Therefore, when tKeima-based protein engineering is used, it is essential to measure not only the oligomeric state but also the fluorescence characteristics.

## 4. Materials and Methods

### 4.1. Sample Preparation

The tKeima codon sequence was optimized for expression in *Escherichia coli*. The corresponding gene was synthesized (Bioneer, Republic of Korea) and inserted into the pET28a vector (Novagen, Madison, WI, USA) between the NdeI and XhoI restriction sites. The corresponding DNA vectors were transformed into *Escherichia coli* BL21(DE3) cells (Novagen, Madison, WI, USA). Cells were grown at 37 °C in LB broth medium containing 50 mg/mL kanamycin. Protein expression was induced by adding isopropyl β-D-thiogalactopyranoside (IPTG) to a final concentration of 0.5 mM at an OD_600_ of approximately 0.8. Cells were incubated at 18 °C for 18 h of shaking at 150 rpm and harvested by centrifugation at 4000 rpm. Cells were resuspended in a buffer containing 50 mM Tris–HCl (pH 8.0) and 200 mM NaCl. Cells were then disrupted by sonication on ice. Cell debris was removed via centrifugation at 13,000 rpm. The supernatant was loaded onto 5 mL His-NTA resin (Qiagen, Valencia, CA, USA) in a column. The resins were washed using a buffer containing 50 mM Tris–HCl (pH 8.0), 200 mM NaCl, and 20 mM imidazole. Proteins were eluted using a buffer containing 50 mM Tris–HCl (pH 8.0), 200 mM NaCl, and 300 mM imidazole. Eluted protein fractions were incubated with thrombin overnight to remove the N-terminal hexahistidine tag. The protein solution was concentrated using a concentrator (Merck Millipore, Burlington, MA, USA) and loaded onto a Sephacryl S-100 column (GE Healthcare, Chicago, IL, USA) in a buffer containing 10 mM Tris–HCl (pH 8.0) and 200 mM NaCl. The eluted protein was concentrated using a concentrator and stored in a refrigerator at 4 °C.

### 4.2. Spectral Analysis

Purified tKeima solution (100 μL) was loaded into a 96-well black polystyrene plate (Sigma-Aldrich, St. Louis, MO, USA). The excitation and emission peaks of the purified tKeima were scanned using a Synergy H1 microplate reader (BioTek, Winooski, VT, USA) at 25 °C. All experiments were performed in triplicate.

### 4.3. Crystallization

The purified tKeima solution (~30 mg/mL) was stored in a refrigerator at 4° C. The tKeima crystals were spontaneously grown in a 1.5 mL tube for 1 day without further crystallization.

### 4.4. Diffraction Data Collection

X-ray diffraction data were collected at the 11C beamline at the Pohang Light Source II (PLS-II, Pohang, Republic of Korea) [42]. The tKeima crystals were transferred onto glass slides using a pipette. A single crystal was fished using a nylon loop and soaked in a cryoprotectant solution containing 50 mM Tris–HCl (pH 8.0), 200 mM NaCl, and 20% (*v*/*v*) glycerol for 10 s. Cryoprotected tKeima crystals were mounted on a goniometer under liquid nitrogen stream at 100 K. Diffraction data were recorded using a Pilatus 6M detector (DECTRIS, Baden–Daettwil, Switzerland). The diffraction data were indexed, integrated, and scaled using HKL2000 [43] program.

### 4.5. Structure Determination

The electron density map was obtained by using the molecular replacement method using the MOLREP [44] program. The crystal structure of dKeima570 (PDB ID: 8YDO) [45] was used as the search model. The model structure was built using the COOT [46] program. Structure refinement was performed using REFMAC5 (version 5.8.0267) [47]. The structural model was validated using the MolProbity (version 4.5.2) [48] server. Structural figures were generated using PyMOL (version 2.4.1) (https://pymol.org/2/; accessed on 1 January 2024). The tetrameric interface of tKeima was analyzed using PDBePISA (version 1.48) [49]. Amino acid sequence alignment was performed using Clustal Omega (version 1.2.4) [50] and visualized using ESPript 3 (version 3.0.10) [51]. The structure factor and coordinates were deposited in the Protein Data Bank (http://rcsb.org, accessed on 1 January 2024) under accession code 8XC6.

## 5. Conclusions

This study enhanced our understanding of the structural characteristics of the large Stokes shift Keima family. Our analysis of the tetrameric interface of tKeima identified key amino acids, among those substituted to create dKeima and mKeima, that interfered with the interface. Additionally, it provides insights into improving novel monomeric Keima or FP by examining the tetrameric interface of tKeima along with its structural features.

## Figures and Tables

**Table 1:** Data collection and refinement statistics for tKeima.

Data Collection	tKeima
X-ray Source	11C beamline, PLS-II
Wavelength (Å)	0.9794
Space group	P22_1_2_1_
Cell dimension a, b, c (Å) α, β, γ (°)	69.88, 83.50, 109.78 90.0, 90.0, 90.0
Resolution (Å)	50.0–3.00 (3.05–3.00)
Unique reflections	13,250 (649)
Completeness (%)	98.9 (99.7)
Redundancy	4.3 (4.6)
I/σ(I)	10.9 (2.1)
R_merge_	0.119 (0.537)
CC1/2	0.981 (0.750)
CC*	0.995 (0.926)
Refinement	
Resolution (Å)	48.21–3.00
R_work_^a^	0.196
R_free_^b^	0.247
R.m.s. deviations	
Bonds (Å)	0.007
Angles (°)	1.451
*B* factors (Å^2^)	
Protein	53.45
Chromophore	72.80
Ramachandran plot	
Favored (%)	95.18
Allowed (%)	4.13
Outliers (%)	0.69
PDB code	8XC6

Values for the outer shell are given in parentheses. ^a^ R_work_ = Σ||F_obs_| Σ |F_calc_||/Σ|F_obs_|, where F_obs_ and F_calc_ are the observed and calculated structure factor amplitudes, respectively. ^b^ R_free_ was calculated as R_work_ using a randomly selected subset of unique reflections not used for structural refinement.

**Table 2:** Interacting residues with the tKeima chromophore.

Chromophore Atom	Residue (Atom)	Molecule A (Å)	Molecule B (Å)
N2	Glu212 (OE1)	3.03	2.41
O2	Arg92 (NH1)	3.48	3.41
O2	Arg92 (NH2)	2.66	2.82
OH	Ser143 (OG)	3.01	2.94
OH	Arg194 (O)	3.69	3.69
CD2	Phe174 (CE2)	4.43	4.66
CG2	Pro60 (CB)	4.39	4.27
CG2	Met94 (CD)	3.45	3.47
CE1	Leu196 (CB)	3.54	3.29
CE2	Met160 (CE)	3.25	3.22
OE1	Gln210(NE2)	3.26	3.10
NE1	Tyr11 (OH)	3.21	3.22

**Table 3:** Tetramer interface of tKiema.

Interface Interaction	Molecule A [atom]	Distance (Å)	Molecule B [atom]	Molecule A [atom]	Distance (Å)	Molecule B [atom]
A–B Hydrogen bond	Glu91 [OE1]	2.91	Asn125 [N]	Asn125 [N]	2.86	Glu91 [OE1]
	Thr103 [OG1]	2.57	Thr103 [OG1]	Asn125 [OD1]	3.50	Thr177 [OG1]
	Thr103 [OG1]	2.65	Ser122 [OG]	Asn125 [ND2]	3.31	Thr93 [OG1]
	Ser122 [O]	3.66	Thr103 [OG1]	Thr177 [OG1]	3.09	Asn125 [OD1]
	Ser122 [OG]	2.77	Thr103 [OG1]			
Interface Interaction	Molecule A [atom]	Distance (Å)	Molecule B* [atom]	Molecule A [atom]	Distance (Å)	Molecule B* [atom]
A–B* Hydrogen bonds	Glu97 [OE1]	2.98	Arg150 [NH1]	Arg150 [NH2]	2.95	Glu97 [OE2]
	Glu97 [OE2]	2.72	Arg150 [NH2]	Asp158 [OD1]	3.46	Arg146 [NH2]
	Pro142 [O]	2.53	Tyr191 [OH]	Asp158 [OD1]	3.18	Arg146 [NH1]
	Thr144 [O]	3.06	Arg146 [NH2]	Tyr159 [O]	2.96	Arg146 [NH1]
	Arg146 [NH1]	2.78	Met160 [O]	His169 [O]	3.05	Arg150 [NH2]
	Arg150 [NH2]	2.78	His169 [O]	Tyr189 [OH]	3.82	Tyr159 [O]
	Arg150 [NH1]	3.15	Glu97 [OE1]	Tyr191 [OH]	2.33	Pro142 [O]
A–B* Salt bridges	Glu97 [OE1]	3.29	Arg150 [NH2]	Arg150 [NH2]	2.95	Glu97 [OE2]
	Glu97 [OE1]	2.98	Arg150 [NH1]	Asp158 [OD1]	3.46	Arg146 [NH2]
	Glu97 [OE2]	2.72	Arg150 [NH2]	Asp158 [OD1]	3.18	Arg146 [NH1]
	Arg150 [NH1]	3.15	Glu97 [OE1]			

## Data Availability

The structure factor and coordinates are deposited in Protein Data Bank (www.rcsb.org, accessed on 10 March 2024) with PDB code 8XC6 (https://www.rcsb.org/structure/8XC6, accessed on 10 March 2024).
